# Intrathyroidal thymic carcinoma initially suspected as a parathyroid tumor: a case report and literature review

**DOI:** 10.3389/fonc.2025.1714302

**Published:** 2025-12-16

**Authors:** Shunyi Huang, Cenwen Liu, Xueying Zeng, Zihan Huang, Feng Lin, Yongyuan Chen, Guanxiang Zhuo, Yunyun Mei, Qing Guan

**Affiliations:** 1 Department of Head & Neck Surgery, Fudan University Shanghai Cancer Center Xiamen Hospital, Xiamen, China; 2 Department of Pathology, Fudan University Shanghai Cancer Center Xiamen Hospital, Xiamen, China; 3 Xinjiang Medical University, Urumqi, China; 4 Department of General Surgery, The First Hospital of Putian City, Putian, China; 5 Fudan University Shanghai Cancer Center Xiamen Hospital, Xiamen, China; 6 Department of Head and Neck Surgery, Fudan University Shanghai Cancer Center, Shanghai, China; 7 Department of Oncology, Shanghai Medical College, Fudan University, Shanghai, China

**Keywords:** intrathyroidal thymic carcinoma, parathyroid tumor, pathology, case report, literature review

## Abstract

Intrathyroidal thymic carcinoma (ITTC) is a rare malignant tumor that presents significant diagnostic and therapeutic challenges. Herein, we present the case of a 35-year-old woman with a right neck mass initially suspected to originate from the parathyroid but ultimately diagnosed as ITTC. This case illustrates the non-specific clinical manifestations of ITTC, the associated diagnostic difficulties, and emphasizes the essential role of pathological examination in diagnosis and guiding management.

## Introduction

Intrathyroidal thymic carcinoma (ITTC), also known as carcinoma showing thymus-like differentiation (CASTLE), is an extremely rare malignancy, accounting for less than 0.15% of all thyroid cancers (1–4). It was first reported and described by Miyauchi et al. as an intraepithelial thymoma of the thyroid gland(ITET) (5). Most of the case reports of the disease originate from Asian countries, especially China and Japan, and the cause is unknown, which may be related to genetics, environment and other factors (2). ITCC typically presents as a slow-growing neck mass, and its nonspecific clinical and imaging features often result in preoperative misdiagnosis as other more common thyroid or parathyroid tumors. Definitive diagnosis depends largely on postoperative histopathological and immunohistochemical analysis, which usually show positive markers such as CD5, CD117, p63, and CK5/6, while thyroglobulin and TTF-1 are negative (6, 7). Given the rarity of ITTC, there is currently no consensus on the best treatment option, but surgical resection remains the mainstay (4, 8). Adjuvant radiotherapy may be considered, especially if the tumor is locally invaded with cervical lymph node metastasis or local recurrence (6, 9).

Herein, we present the case of a 35-year-old woman with a right neck mass that was initially misdiagnosed as a parathyroid tumor. The diagnosis of ITTC was confirmed only after surgical resection and immunohistochemical examination. The patient eventually underwent surgery followed by adjuvant radiotherapy and remained disease-free during the follow-up period. This case highlights the diagnostic challenges of ITTC and emphasizes the essential role of immunohistochemistry in achieving an accurate diagnosis and guiding appropriate management.

## Case report

**Figure 1:**
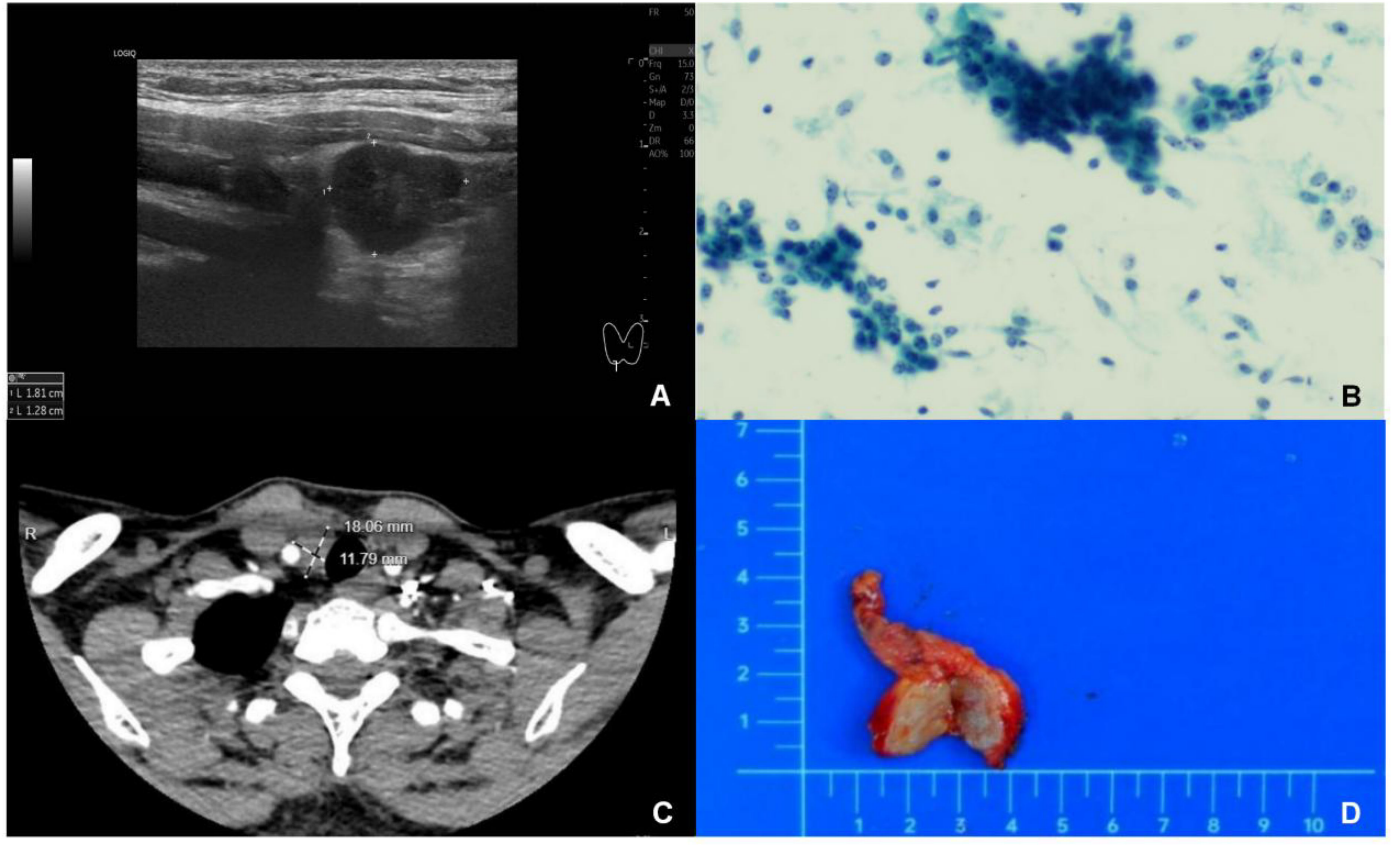
Intrathyroidal thymic carcinoma. **(A)**: Ultrasonography revealed a solid mass measuring 18×13 mm located beneath the right thyroid gland. The mass exhibited hypoechogenicity, with well - defined margins and a regular shape. **(B)** Fine-needle aspiration biopsy showed cells arranged in clusters, sheets, and scattered. They were crowded and disorganized, with round, oval, or short - spindle shapes. Most cells were similar, but a few had enlarged, atypical nuclei with 1–3 distinct nucleoli and nuclear displacement. **(C)** CT scans showed the mass was hypodense with clear margins. It had mild to moderate enhancement after contrast injection, no obvious calcification, and was adjacent to the right common carotid artery and trachea. **(D)** Gross examination revealed a solid tumor with clear margins, regular shape, grayish - yellow cut surface, and no obvious capsule.

A 35-year-old woman was admitted after a right neck mass was detected during a routine health examination. Ultrasonography revealed a well-defined, regular hypoechoic nodule measuring approximately 18 × 13 mm inferior to the right thyroid lobe, with internal blood flow signals on color doppler flow imaging (CDFI) (**Figure 1A**), raising suspicion of parathyroid origin. Fine-needle aspiration cytology (FNAC) suggested malignancy (**Figure 1B**), with a malignant potential of Bethesda Category V. Molecular testing showed no BRAF V600E mutation in exon 15. Contrast-enhanced CT subsequently demonstrated a nodule inferior to the right thyroid lobe, likely of thyroid origin, in close proximity to the right common carotid artery and trachea (**Figure 1C**). PET/CT suggested a malignant tumor inferior to the lower pole of the right thyroid lobe, possibly of parathyroid origin, though thyroid origin could not be excluded. No concurrent mediastinal tumor was detected, and the patient had no personal or family history of thymic tumors. Preoperative thyroid function, parathyroid hormone (PTH), calcitonin, serum calcium, and phosphorus levels were all within normal ranges.

Eventually, the patient underwent surgical resection of the mass. Intraoperatively, a firm, ill-defined tumor was identified, invading adjacent structures, including the thymus and strap muscles. Frozen section analysis suggested poorly differentiated carcinoma, and a right central compartment (Level VI) lymph node dissection was subsequently performed.

**Figure 2:**
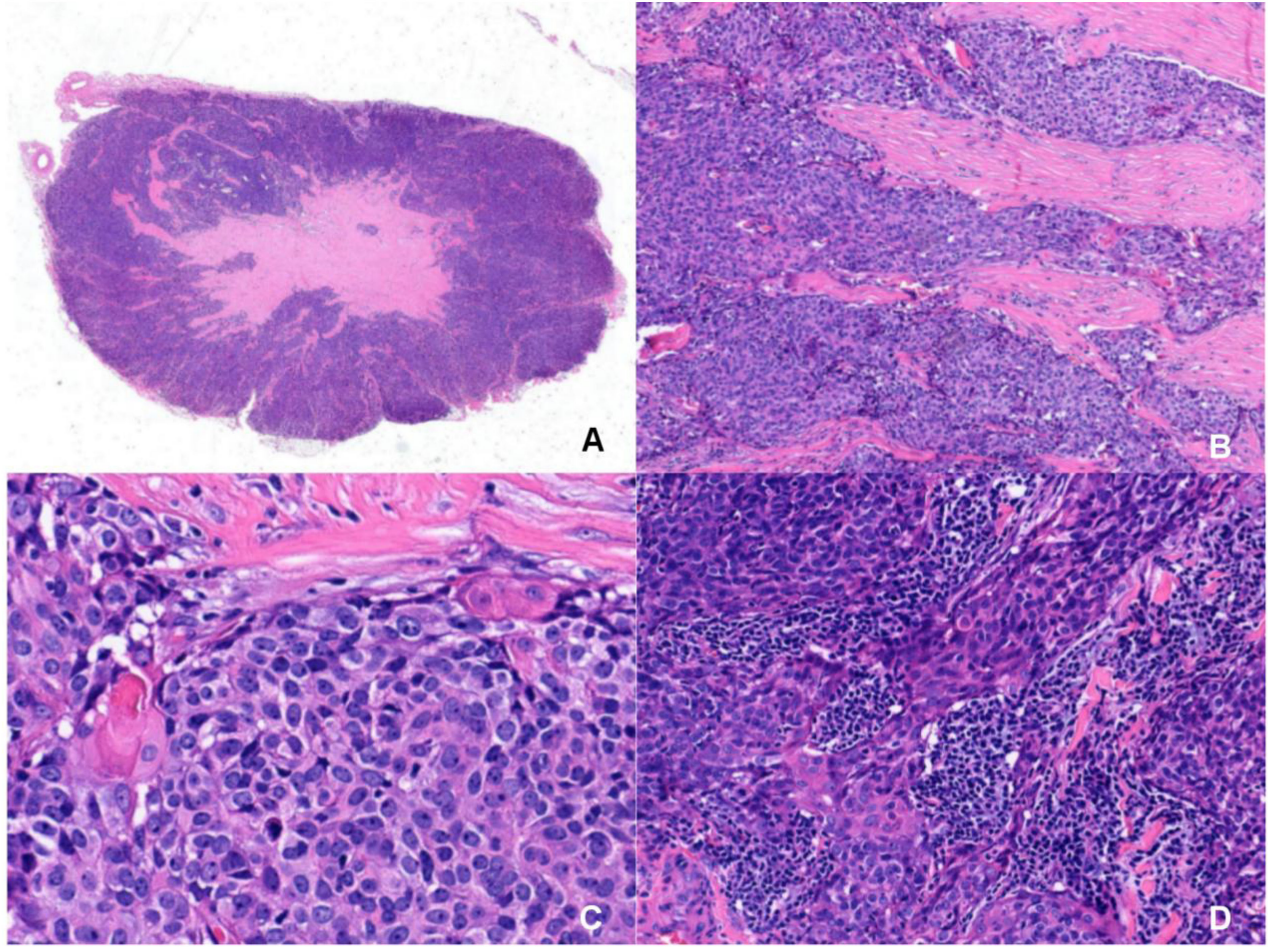
Microscopic morphology of intrathyroidal thymic carcinoma with HE staining. **(A)** The tumor has a relatively circumscribed border and the stroma show extensive collagenization and sclerosis(Magnification: 6×). **(B)** The tumor cells show infiltrative growth, arranged in sheets and islands, separated by dense fibrous tissue of varying widths(Magnification: 200×). **(C)** The tumor cells are polygonal with eosinophilic cytoplasm, featuring large vesicular or hyperchromatic nuclei with prominent nucleoli. The mitotic figures are visible. In some areas, squamous differentiation are observed(Magnification: 400×). **(D)** Lymphocytic infiltration is observed around the tumor cell nests(Magnification: 200×).

**Figure 3:**
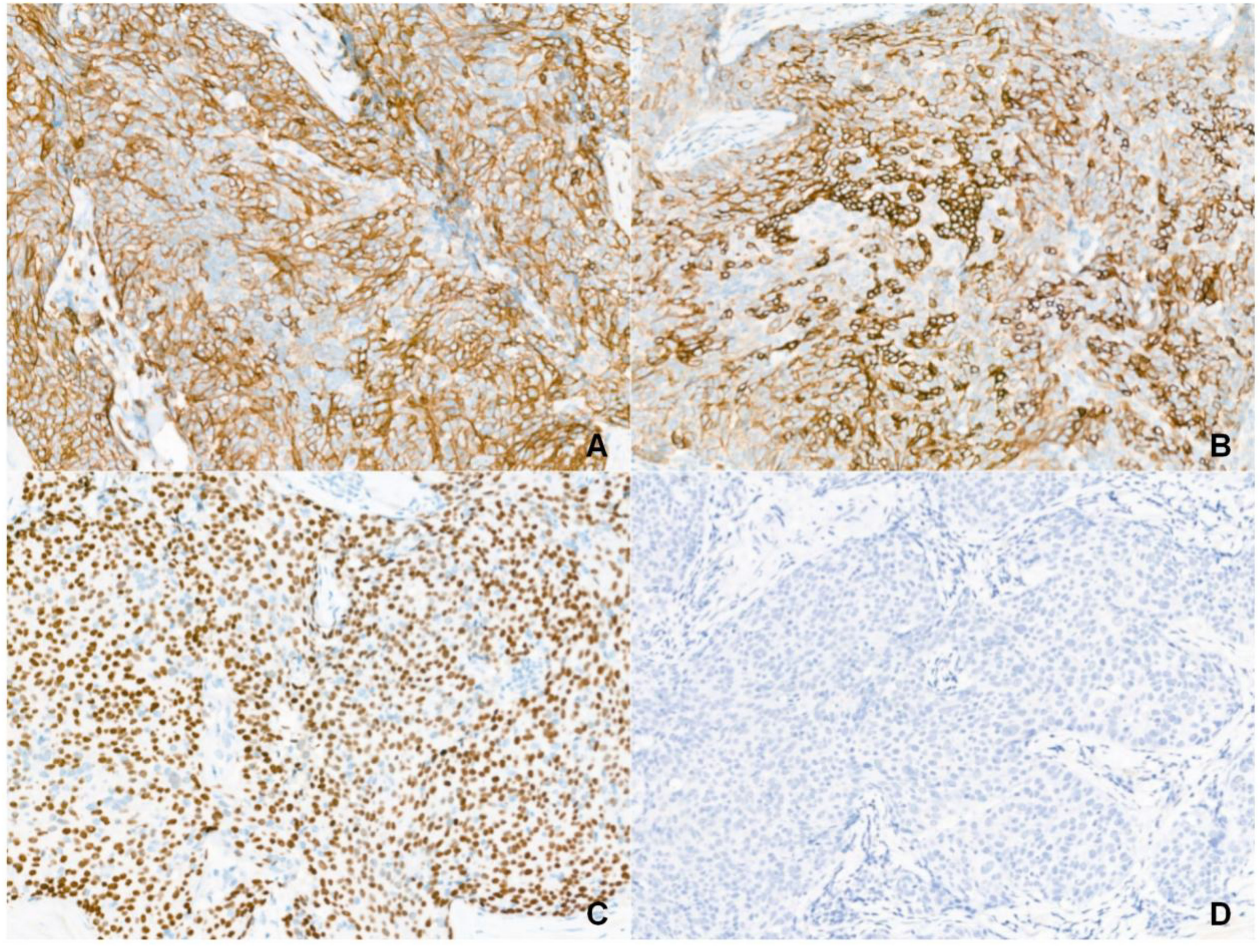
Immunophenotype of intrathyroidal thymic carcinoma (Magnification: 200×). **(A)** CD5 is diffuse positive. **(B)** CD117 is diffuse positive. **(C)** P63 is diffuse positive. **(D)** TTF-1 is negative.

Gross examination of the resected specimen (3.0 × 2.0 × 1.5 cm) revealed a gray-yellow, firm, well-circumscribed mass (**Figure 1D**). Six lymph nodes were retrieved from the right Level VI dissection. Histological examination revealed that the tumor had a relatively circumscribed border, with stroma exhibiting extensive collagenization and sclerosis (**Figure 2A**). The tumor cells demonstrated infiltrative growth, arranged in sheets and islands separated by dense fibrous tissue of varying thickness (**Figure 2B**). The cells were polygonal with eosinophilic cytoplasm and displayed large vesicular or hyperchromatic nuclei with prominent nucleoli. Besides, mitotic figures were readily observed, while in some areas, squamous differentiation was noted (**Figure 2C**). Of note, lymphocytic infiltration was present around the tumor cell nests (**Figure 2D**). Immunohistochemically, the tumor cells were strongly and diffusely positive for CD5, CD117, P63, CK5/6, and P40, with additional positivity for synaptophysin (Syn) and INSM1. In contrast, TTF-1, TdT, and GATA3 were negative (**Figures 3A–D**). The Ki-67 labeling index was approximately 20%, and p53 expression was of the wild-type pattern. The final diagnosis was right intrathyroidal thymic carcinoma, measuring 2.3 × 1.5 × 1.0 cm, without evidence of lymphovascular invasion. All examined lymph nodes (peritumoral: 0/1; Level VI: 0/6) were negative for metastasis.

The patient recovered well and was discharged without complications. She subsequently received postoperative radiotherapy (50 Gy in 25 fractions) to the right paratracheal region. Follow-up ultrasound and neck/chest CT at three months revealed no evidence of recurrence. This case underscores the importance of including ITTC in the differential diagnosis of neck masses, particularly when imaging and cytology are inconclusive. Accurate diagnosis relies on comprehensive histopathological and immunohistochemical evaluation, while early surgical intervention and thorough pathological assessment remain essential for the effective management of this rare and diagnostically challenging malignancy.

Therefore, we have collected the relevant articles published on pubmed in the past 10 years, as shown in **Table 1**.

**Table 1:** ITTC-related reports on pubmed in the past 10 years.

Year	Author	Number of cases	Surgery	Extrathyroid invasion	Adjuvant therapy	Outcome
2016	Liu et al (10).	1	Tumor resection+partial tracheotomy	trachea,RLN	–	NER 12m
2016	Wu et al (11).	1	Total thyroidectomy+ CLND+tangential resection of the trachea	trachea, esophagus	–	NER 8m
2017	Lominska et al (12).	1	Total thyroidectomy+CLND	–	RT	NER 6yr
2018	Inoue et al (7).	1	Subtotal thyroidectomy+bilateral CLND	strap muscle, parathyroid gland	RT	NER 5yr
2018	Rajeshwari et al (13).	1	Total thyroidectomy+CLND	–	–	–
2019	Chung et al (14).	1	Total thyroidectomy	–	RT	–
2019	Fung et al (15).	1	Total thyroidectomy+selective neck dissection	trachea,RLN	RT	NER 18m
2019	Ren et al (16).	1	Thyroid lobectomy+CLND	–	–	–
2019	Tran et al (17).	1	Total thyroidectomy+CLND	esophagus, trachea, RLN	–	–
2020	Jiang et al (18).	1	Tumor resection+radical left neck dissection	–	RT	NER 1yr
2021	Kimura et al (19).	1	Hemithyroidectomy+CLND	–	–	NER 10yr
2022	Kimura et al (20).	1	Total thyroidectomy+CLND+lateral cervical ND	esophagus, trachea, RLN	RT	NER 5yr
2022	Kuroki et al (21).	1	Total thyroidectomy+ CLND+cervical tracheal resection	trachea,RLN	CCRT after fourth surgery	LN recurrence at 35m,45m,62m,77m,alive 90m
2022	Stanciu et al (22).	1	Total thyroidectomy+CLND	esophagus	–	NER 2yr
2023	Cui et al (23).	1	Total thyroidectomy+CLND	strap muscle	RT	NER 4yr
2023	Zhao et al (24).	2	Total thyroidectomy+CLND;Lobectomy+CLND	trachea;-	–	NER 3yr; NER 12m
2024	Anila et al (25).	1	Total thyroidectomy+CLND+lateral cervical ND	esophagus, RLN	RT	–
2024	Chen et al (26).	1	none	none	–	Lung metasis after 4yr,alive 6yr
2024	Hsu et al (27).	1	Total thyroidectomy+CLND	trachea,RLN	–	–
2024	Lee Chun Yun et al (28).	1	Total thyroidectomy+CLND	esophagus, trachea, RLN	RT	–
2025	Nagaoka et al (29).	1	Total thyroidectomy+CLND	RLN	RAI	NER 5yr
2025	Wang et al (30).	1	Biopsy	RLN	RT	–
2025	Xu et al (31).	1	Partial thyroidectomy + cervical LN biopsy	trachea,RLN	–	–

ND, neck dissection; CLND, central lymph node dissection; RLN, recurrent laryngeal nerve; RND, radical neck dissection; RT, radiotherapy; NER, no evident recurrence; RAI, radioactive iodine; yr, years; m, months.

## Discussion

ITTC is an exceedingly rare tumor thought to arise from ectopic thymic tissue or branchial pouch remnants (30, 32–35). It shows a female predominance and primarily affects middle-aged individuals (40–50 years) (3, 16, 36). Most ITTCs are located at the lower pole of the thyroid or in the parathyroid region near the inferior thyroid pole, although rare cases have been reported in the parotid gland, submandibular gland, or parapharyngeal space (1, 6). Patients may be asymptomatic or present with a slow-growing, painless neck mass, while symptoms such as hoarseness, dysphagia, or dyspnea may occur when there is local invasion or lymph node involvement (3, 4, 37).

Nonetheless, ITTC lacks specific imaging characteristics. Ultrasound, often the first-line modality, typically reveals a solid, heterogeneous, ill-defined hypoechoic mass without calcifications or cystic changes, while CT imaging demonstrates an ill-defined, mildly enhancing soft-tissue density mass (38). Conversely, MRI provides limited diagnostic value, while PET/CT can be helpful in assessing distant metastases, although additional clinical data are needed to clarify its role (23, 37, 39). FNAC is important for preoperative evaluation but demonstrates low sensitivity (1.5–8.3%) for ITTC, with high false-negative rates, and cannot reliably distinguish it from squamous cell carcinoma, poorly differentiated carcinoma, anaplastic thyroid carcinoma, or thymic carcinoma (23, 37, 40). Thus, ITTC’s rarity and non-specific cytology make preoperative diagnosis particularly challenging. The differential diagnosis typically includes anaplastic thyroid carcinoma, squamous cell carcinoma, medullary thyroid carcinoma, metastatic head and neck cancers, and low-grade lymphoma (6, 30, 41). The non-specific findings in this case illustrate this diagnostic difficulty. When FNAC is inconclusive, core needle biopsy with immunocytochemical analysis may provide additional diagnostic value.

In the present case, intraoperative frozen section analysis suggested only poorly differentiated carcinoma, complicating surgical decision-making. Previous reports have noted that frozen sections can be misleading in ITTC (42). Most ITTC cases are diagnosed postoperatively. Histologically, ITTC can be subclassified into keratinizing squamous cell carcinoma, non-keratinizing basaloid carcinoma, and neuroendocrine carcinoma (6). Tumor nests are separated by fibrous stroma, and the cells are generally spindle-shaped or polygonal with prominent nucleoli and indistinct borders. Moreover, squamous differentiation with lymphoplasmacytic infiltration is commonly observed. Histological differential diagnoses include anaplastic carcinoma, lymphoma, insular carcinoma of the thyroid, and lymphoepithelioma-like thymic carcinoma (41).

Immunohistochemistry is pivotal for diagnosing ITTC. Tumor cells are strongly positive for CD5 and CD117, and often express p63, high molecular weight cytokeratin (HMWCK), wild-type p53, Bcl-2, S100A9, CEA, PAX8, EGFR, reticulocalbin, Mcl-1, and GLUT-1. ITTC is consistently negative for thyroid markers, including calcitonin, TTF-1, and thyroglobulin (6, 23, 33). A low Ki-67 index (typically 10–30%, usually < 20%) helps distinguish ITTC from anaplastic or squamous cell carcinoma, which often shows Ki-67 > 50% (33). The neuroendocrine subtype may also express Syn, CgA, or NSE (6, 16, 30). Notably, monoclonal PAX8 and CD5 antibodies are useful in differentiating ITTC from poorly differentiated thyroid carcinoma (PDTC) and squamous cell carcinoma (SCC) (43). EBER *in situ* hybridization (ISH) is generally negative in ITTC, unlike mediastinal thymic carcinoma, suggesting no association with EBV. Meanwhile, TERT promoter mutations have been identified in some ITTC cases but not in mediastinal thymic carcinomas, which may underlie their biological differences (44). Strong CK5/6 and P40 expression, commonly seen in squamous cell carcinoma, must be interpreted in conjunction with CD5 positivity to differentiate ITTC from primary squamous cell carcinoma of the thyroid (PSCCT) (45). The negative GATA3 and INSM1 staining in this case excluded parathyroid lesions and medullary thyroid carcinoma (46). Meanwhile, postoperative findings contradicted the initial preoperative suspicion of a parathyroid tumor. Although ITTC should be considered in the preoperative differential diagnosis, its rarity often leads to it being overlooked.

Surgical resection is the primary treatment for ITTC (3, 8, 23, 43). Although generally considered low-grade, ITTC can be locally aggressive, making complete resection and close follow-up essential. Radical surgery, including lobectomy or total thyroidectomy with excision of involved structures and central compartment dissection, is commonly preferred (3, 23, 40). Thyroidectomy is indicated in cases of extrathyroidal extension (ETE), lymph node involvement, or distant metastasis (23). Importantly, lymph node dissection has been shown to improve survival in ETE-positive cases, and prophylactic dissection may help reduce local recurrence (8, 32). Reviewing the relevant cases reported in the past 10 years, gland resection, especially total thyroidectomy, is considered to be a choice that helps local lesions and prognosis, and central lymph node dissection should also be an important part of surgical treatment.

Here, central compartment dissection was performed without thyroidectomy due to the well-defined intraoperative tumor boundary and inconclusive frozen section analysis. The need for completion thyroidectomy or a more extensive neck dissection generally depends on follow-up findings. For our patient, the absence of lymphovascular invasion suggests a favorable prognosis. Postoperative radiotherapy can reduce the risk of recurrence, particularly in cases of incomplete resection, ETE, or lymph node metastasis (6, 32, 37), and may also improve survival (8). For locally advanced, inoperable tumors, radiotherapy, with or without chemotherapy, has been shown to be effective (3, 30). In addition, salvage surgery or radiotherapy may benefit patients with local recurrence (3, 6). Chemotherapy can be considered for widely invasive or metastatic disease, although supporting evidence is limited (37, 40). Treatment with Lenvatinib has shown efficacy for advanced thymic carcinoma, but data specific to ITTC are scarce (40). On the other hand, immune checkpoint inhibitors have shown potential for treating metastatic ITTC (47). ITTC is a low-grade malignancy with an indolent behavior, but it can invade locally and metastasize to lymph nodes, lungs, liver, bone, or brain, with reported 5- and 10-year survival rates of 90% and 82%, respectively (34). Patients without nodal involvement have better outcomes, while the presence of lymph node metastasis and ETE are important prognostic factors (23). Nevertheless, global data remain limited, and no standardized management guidelines currently exist.

## Limitations

This study has several limitations that should be taken into account. Preoperative suspicion of a parathyroid tumor, despite normal PTH levels, led to omission of MIBI scintigraphy. Surgical management consisted of mass resection and central compartment dissection without thyroidectomy or comprehensive neck dissection, although the patient was diagnosed with ITTC. Follow-up data are limited to the short term, and longer monitoring is required to fully assess prognosis. Furthermore, additional cases are needed to better define survival outcomes and establish optimal management strategies for ITTC.

## Conclusion

ITTC is a rare and diagnostically challenging malignancy that requires a high index of suspicion and comprehensive immunohistochemical analysis for accurate diagnosis. Surgical resection remains the mainstay of treatment, with adjuvant radiotherapy aiding to reduce recurrence risk. ITTC’s generally favorable prognosis underscores the importance of early detection and appropriate surgical management. Taken together, this case contributes to the literature on ITTC and highlights the crucial role of a multidisciplinary approach in managing such rare tumors.

## Data Availability

The original contributions presented in the study are included in the article/supplementary material. Further inquiries can be directed to the corresponding authors.
